# Supplementary material for: Potential of a Bead-Based Multiplex Assay for SARS-CoV-2 Antibody Detection
Source: Biology (Basel). 2024 Apr 18;13(4):273. doi: 10.3390/biology13040273 (PMC11047883; doi:10.3390/biology13040273)
Supplement: Supplementary file 1 [file biology-13-00273-s001.zip › biology-2927437-supplementary.pdf]

Potential of a bead-based multiplex assay for SARS-CoV-2 antibody detection

Karla Rottmayer[1], Mandy Schwarze[2,3], Jassoy Christian[4], Ralf Hoffmann[2,3], Henry Loeffler-Wirth[5] and Claudia Lehmann[1] *

Supplementary figures

(a)

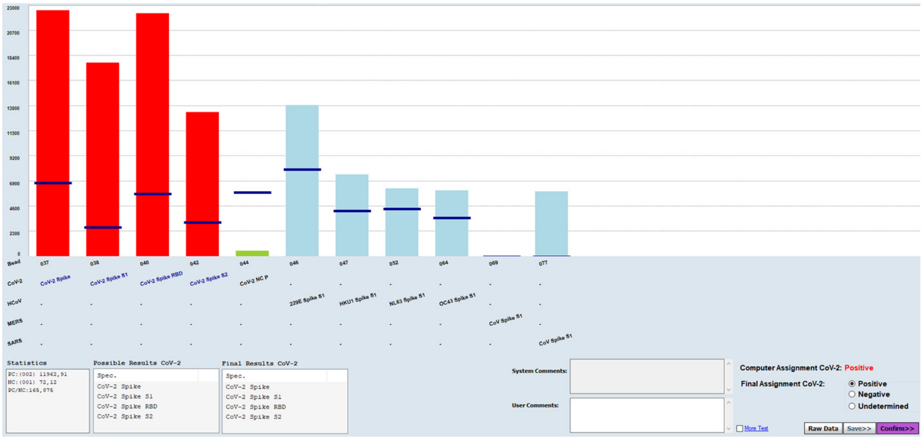

(b)

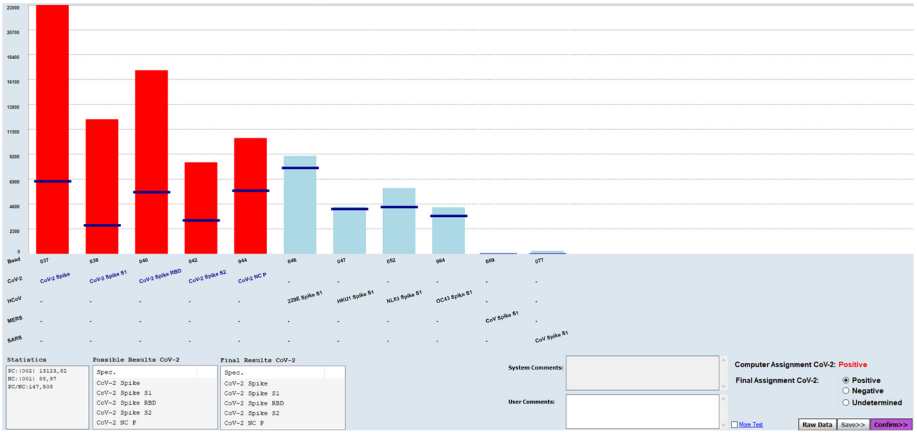

(c)

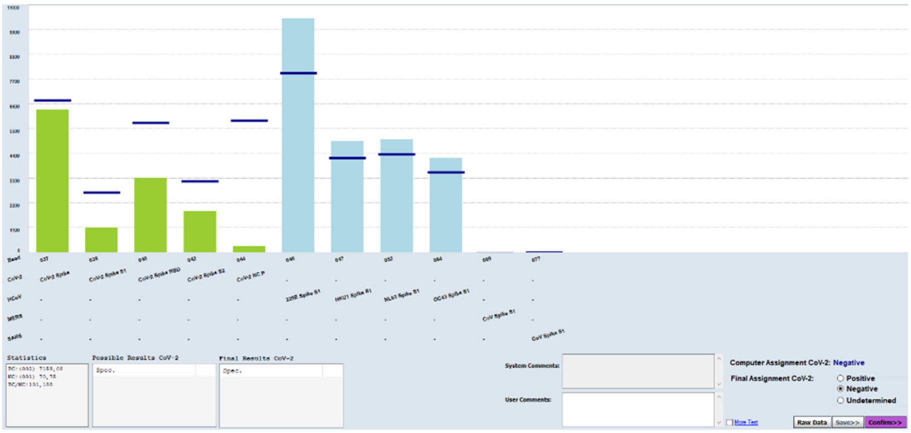

Figure S1. Data analysis using HLA-Fusion™ software. (a) A positive serum with SARS-CoV-2 positive reactions (red bars) and negative bead reaction for NCP. (b) A positive serum with SARS-CoV-2 positive reactions against all five SARS-CoV-2 beads (red bars). (c) A negative serum without SARS-CoV-2 positive reactions (green bars). The blue bars indicate the reactions against endemic coronavirus antigens, such as S1 domain, MERS-CoV and SARS-CoV. The dark blue horizontal lines mark the bead-specific cut-off.

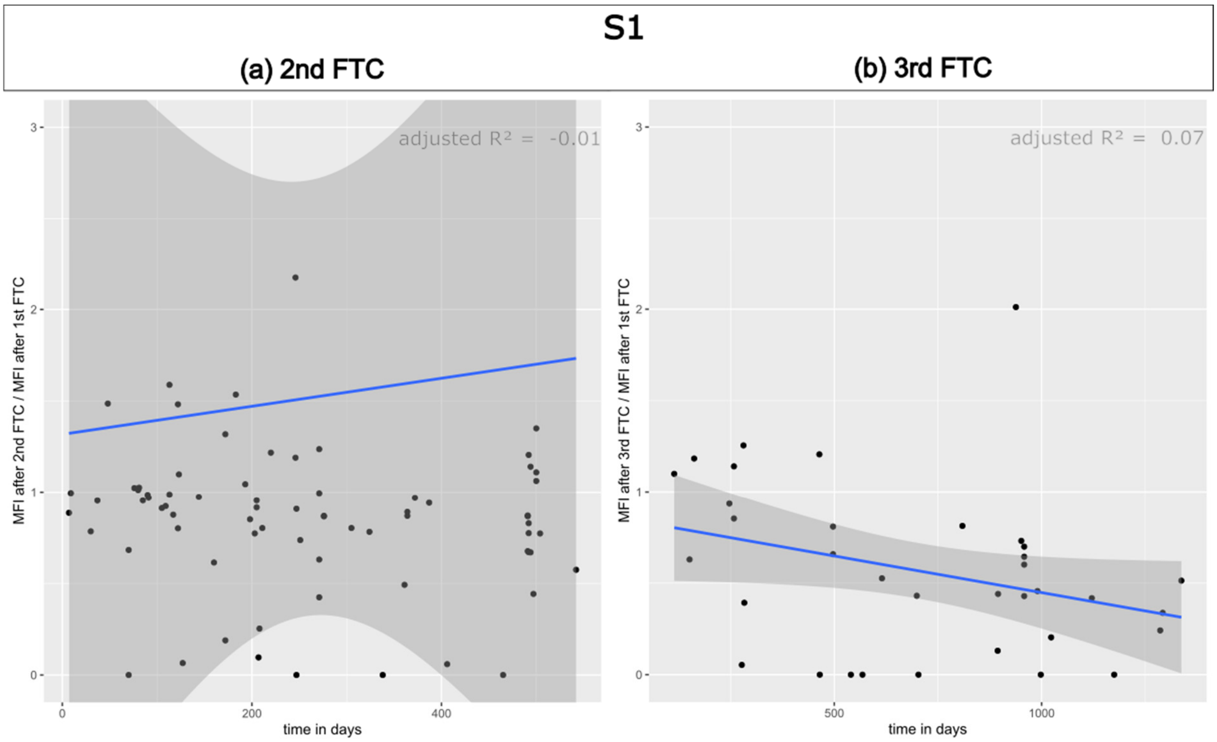

Figure S2. The correlation between the percentage MFI values of the S1 antibody and the storage duration was examined. The results are shown in (a) for the sera after the second freeze-thaw cycle and in (b) for the sera after the third freeze-thaw cycle. The adjusted R^2 values are displayed in the top right corner.

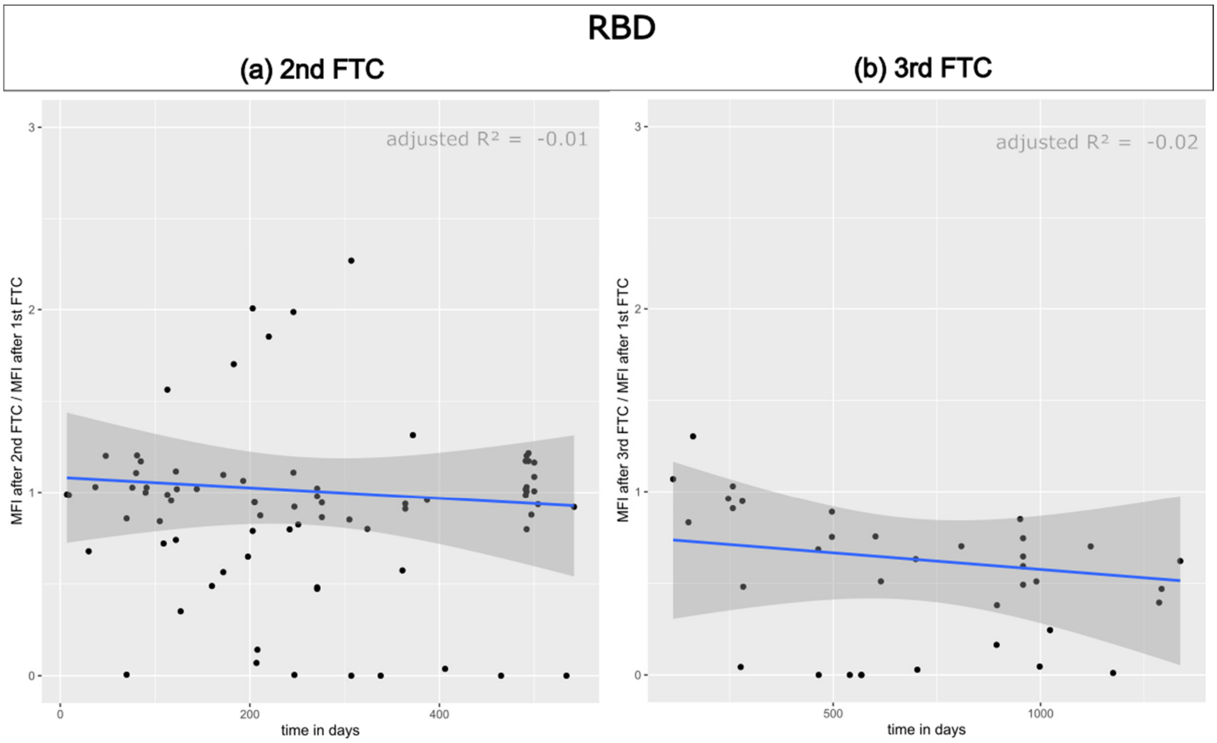

Figure S3 The correlation between the percentage MFI values of the RBD antibody and the storage duration was examined. The results are shown in (a) for the sera after the second freeze-thaw cycle and in (b) for the sera after the third freeze-thaw cycle. The adjusted R^2 values are displayed in the top right corner.

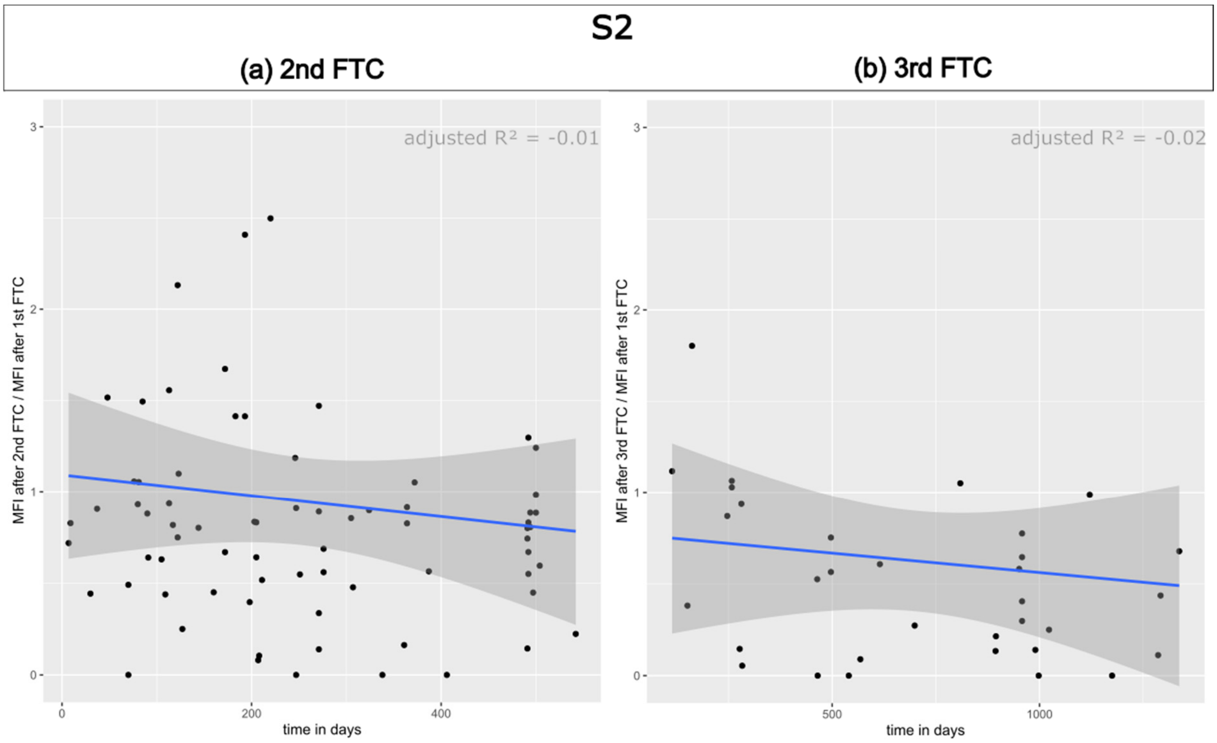

Figure S4. The correlation between the percentage MFI values of the S2 antibody and the storage duration was examined. The results are shown in (a) for the sera after the second freeze-thaw cycle and in (b) for the sera after the third freeze-thaw cycle. The adjusted R^2 values are displayed in the top right corner.

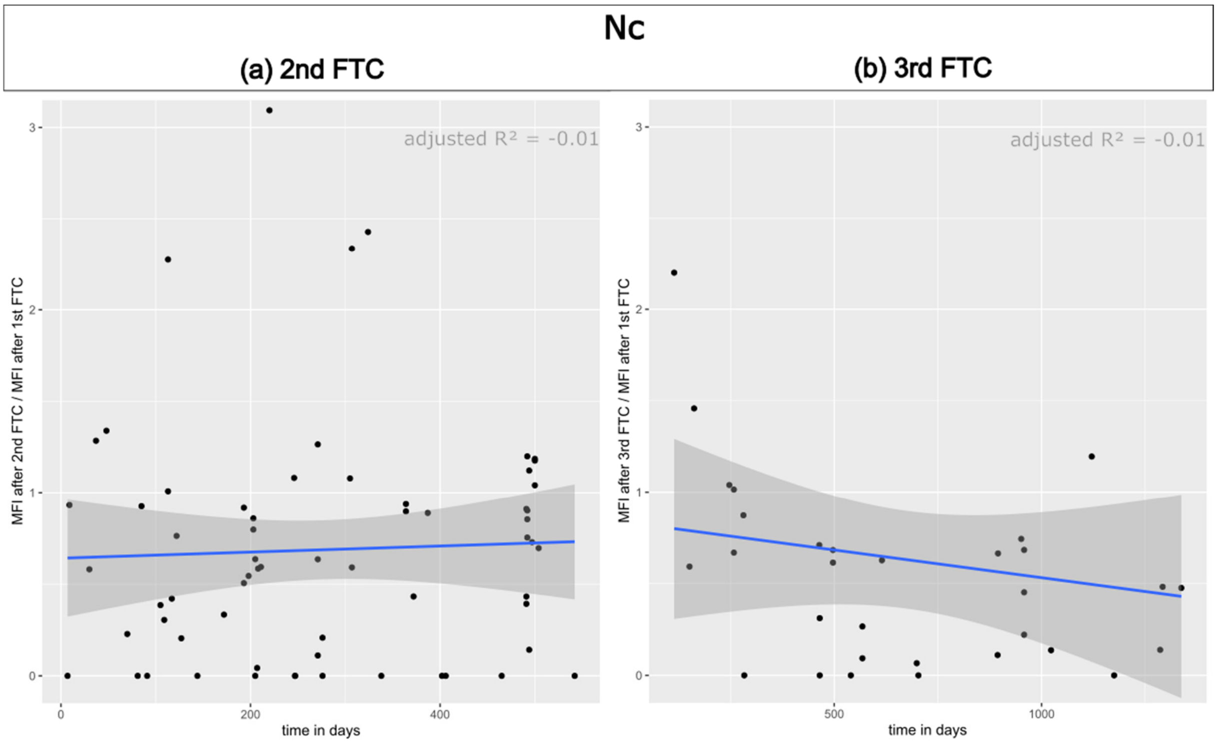

Figure S5. The correlation between the percentage MFI values of the Nc antibody and the storage duration was examined. The results are shown in (a) for the sera after the second freeze-thaw cycle and in (b) for the sera after the third freeze-thaw cycle. The adjusted R^2 values are displayed in the top right corner.
